# Human Thymic CD10^+^ PD-1^+^ Intraepithelial Lymphocyte Precursors Acquire Interleukin-15 Responsiveness at the CD1a^–^ CD95^+^ CD28^–^ CCR7^–^ Developmental Stage

**DOI:** 10.3390/ijms21228785

**Published:** 2020-11-20

**Authors:** Lore Billiet, Glenn Goetgeluk, Sarah Bonte, Stijn De Munter, Laurenz De Cock, Melissa Pille, Joline Ingels, Hanne Jansen, Karin Weening, Filip Van Nieuwerburgh, Tessa Kerre, Tom Taghon, Georges Leclercq, Bart Vandekerckhove

**Affiliations:** 1 Department of Diagnostic Sciences, Ghent University, 9000 Ghent, Belgium; Lore.Billiet@ugent.be (L.B.); Glenn.Goetgeluk@ugent.be (G.G.); Stijn.DeMunter@ugent.be (S.D.M.); Melissa.Pille@ugent.be (M.P.); Joline.Ingels@ugent.be (J.I.); Hanne.Jansen@UGent.be (H.J.); Karin.Weening@ugent.be (K.W.); Tessa.Kerre@ugent.be (T.K.); Tom.Taghon@ugent.be (T.T.); Georges.Leclercq@ugent.be (G.L.); 2 Cancer Research Institute Ghent (CRIG), 9000 Ghent, Belgium; SarahM.Bonte@ugent.be (S.B.); Laurenz.DeCock@ugent.be (L.D.C.); Filip.VanNieuwerburgh@ugent.be (F.V.N.); 3 Department of Internal Medicine and Pediatrics, Ghent University, 9000 Ghent, Belgium; 4 Department of Biomolecular Medicine, Ghent University, 9000 Ghent, Belgium; 5 Department of Pharmaceutics, Ghent University, 9000 Ghent, Belgium

**Keywords:** intraepithelial lymphocytes, CD8αα-positive T cells, T cell activation

## Abstract

Human thymic CD8αα^+^ CD10^+^ PD-1^+^ αβ T cells selected through early agonist selection have been proposed as the putative thymic precursors of the human CD8αα^+^ intestinal intraepithelial lymphocytes (IELs). However, the progeny of these thymic precursor cells in human blood or tissues has not yet been characterized. Here, we studied the phenotypical and transcriptional differentiation of the thymic IEL precursor (IELp) lineage upon in vitro exposure to cytokines prominent in the peripheral tissues such as interleukin-15 (IL-15) and the inflammatory cytokines interleukin-12 (IL-12) and interleukin-18 (IL-18). We showed that only the CD1a^−^ fraction of the CD10^+^ PD-1^+^ IELp population was able to proliferate with IL-15, suggesting that this subset had acquired functionality. These cells downregulated PD-1 expression and completely lost CD10 expression, whereas other surface markers such as CD95 and CXCR3 remained highly expressed. RNA-seq analysis of the IL-15-cultured cells clearly showed induction of innate-like and effector genes. Induction of the cytotoxic machinery by the CD10^+^ PD-1^+^ population was acquired in the presence of IL-15 and was further augmented by inflammatory cytokines. Our data suggest that only the CD1a^−^ CD10^+^ PD-1^+^ population exits the thymus and survives in the periphery. Furthermore, PD-1 and CD10 expression is not an intrinsic property of this lineage, but rather characterizes a transient stage in differentiation. CD95 and CXCR3 expression combined with the absence of CD28, CCR7, and CD6 expression might be more powerful markers to define this lineage in the periphery.

## 1. Introduction

Conventional adaptive CD4^+^ and CD8^+^ αβ T cells are the main T cell populations generated in the thymus [1]. After stringent selection, a small percentage of these T cells emigrate from the thymus. These naive cells recirculate between blood and secondary lymphoid organs including lymph nodes, spleen, Peyer’s patches, awaiting antigen encounter. Since a particular T cell is unlikely to ever encounter an antigen, most conventional T cells recirculate as dormant, functionally inactive cells [2]. Besides conventional T cells, several lineages of so-called unconventional T cell populations are generated concurrently. These T cells resemble innate cells as some of these have a restricted specificity and as the cells are generally in a more active functional state. Besides the unconventional γδ T cells, alternative TCRαβ^+^ lineages, namely regulatory T (Treg), mucosal-associated invariant T (MAIT), natural killer T (NKT) and intraepithelial lymphocyte (IEL) T cells are generated in the thymus. Treg cells are a distinct subset of CD4^+^ T cells diverted upon high-affinity encounter of self-antigens in the thymic medulla. These cells are polyclonal, autoreactive and safeguard immune tolerance [3]. MAIT and NKT cells are innate-like T cells with a restricted specificity and a semi-invariant T cell receptor (TCR), emigrating the thymus as activated, functional cells [4]. Similarly, precursors of IELs leave the thymus as activated T cells, but in contrast to MAIT and NKT cells, this population expresses polyclonal TCRs [5,6].

In addition to the induced memory TCRαβ^+^ CD4^+^ and TCRαβ^+^ CD8αβ^+^ cells and the TCRγδ^+^ cells, TCRαβ^+^ CD4^–^ CD8α^+^ CD8β^–^ intestinal intraepithelial lymphocytes (CD8αα IELs) form a prominent thymus-derived T cell population that guards the gut epithelium [7]. In recent years, the development of CD8αα IELs has been investigated predominantly in mice. Thymic IEL precursors (IELps) divert from conventional T cell development upon high-affinity TCR interaction at the CD4 CD8 double-positive (DP) stage. Although high-affinity TCR interaction usually leads to clonal deletion, some precursor T cells are induced to differentiate into CD4 CD8 double-negative (DN) CD3^+^ T cells that express gut homing receptors [6]. Ruscher et al. divide this mature DN population into two distinct IELp populations with each a polyclonal TCR and differential major histocompatibility complex (MHC) restriction. Both IELp populations can seed the murine gut, where they express CD8αα [8]. Type A thymic IELps include self-reactive PD-1^+^ thymocytes that show hallmarks of thymocytes undergoing elevated TCR signaling and strong signs of agonist selection [6,8]. Type B thymic IELps consist of a smaller T-bet^+^ NK1.1^+^ population. These seed the intestine in a restricted time period early in life during which they display thymic emigration markers such as KLF2 and S1PR1 and the mucosal-homing integrin α_4_β_7_. With progressing age, type B IELps lose these characteristics and seem to become thymus-resident. Type A IELps present a constant emigration-competent and mucosal-homing phenotype while showing a more slow but steady gut entry and eventually become the predominant type in the adult murine gut [8,9]. In contrast, a recent report using single-cell RNA-sequencing analysis presented evidence that all T-bet^+^ IELps progress through a PD-1^+^ stage before upregulating T-bet, all arising from one common thymic precursor population [10]. In conclusion, CD8αα^+^ IEL development remains enigmatic, as well as its main function and mode of action.

Our research lab previously described the likely precursors of IELs in the human thymus. These human IELps present in the thymus with a CD10^+^ PD-1^+^ phenotype and, unlike in mice, express both CD8αα and CD8αβ [11]. These agonist-selected CD10^+^ PD-1^+^ T cells have diverse TCR repertoires, characterized by preferential usage of T cell receptor alpha joining (*TRAJ*)-proximal Vα gene segments and T cell receptor alpha variable (*TRAV*)-proximal Jα gene segments [11]. This characteristic repertoire was recently also reported for type A IELps in mice. Furthermore, a high percentage of TCRs with a high hydrophobic index and cysteine index were observed in the human and murine IELps, in line with the self-reactivity observed in the IELp population [12,13].

Here, the human TCRαβ^+^ CD8αα^+^ CD10^+^ PD-1^+^ IELp population was further studied. It was assessed whether immature and mature thymic subpopulations could be identified and whether specific markers could be defined that were stably expressed and possibly lineage defining. Furthermore, the proliferative and cytotoxic capacity of the population was investigated.

## 2. Results and Discussion

### 2.1. CD1a Expression Marks the Immature CD10^+^ PD-1^+^ Fraction

**Figure 1:**
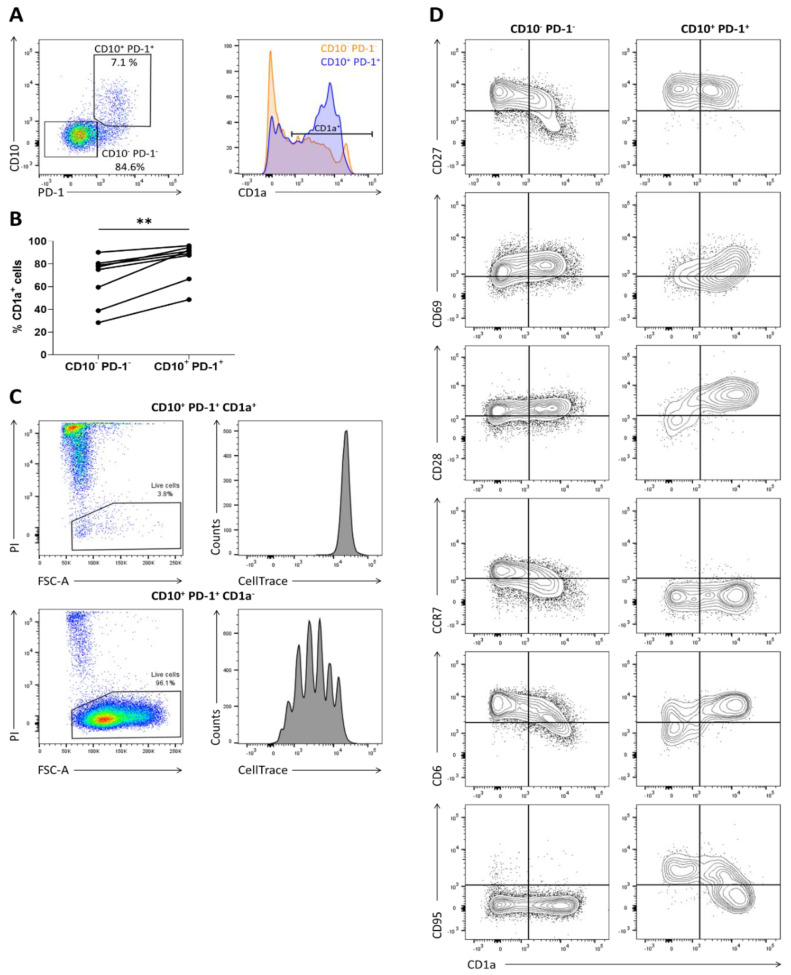
CD1a marks the immature CD10^+^ PD-1^+^ cells incapable of proliferating in the presence of IL-15. (**A**) CD1a expression on CD10^−^ PD-1^−^ and CD10^+^ PD-1^+^ T cells in human postnatal thymus, gated on TCRγδ^–^ CD3^+/low^ CD4^–^ CD8α^+^; (**B**) percentage of CD1a^+^ cells in each population in 8 postnatal thymuses. Connected values correspond to paired populations of the same biological replicate. Wilcoxon matched-pairs signed-rank test was used to assess the statistically significant difference in CD1a expression between the CD10^−^ PD-1^−^ and CD10^+^ PD-1^+^ population. *p*-value < 0.01 (**); (**C**) proliferation and viability assessed by CellTrace Violet dye dilution and propidium iodide (PI) exclusion in CD10^+^ PD-1^+^ CD1a^+^ and CD10^+^ PD-1^+^ CD1a^−^ cells isolated from human postnatal thymus after 5 days of incubation in the presence of IL-15 (10 ng/mL). Data are representative of at least three biological replicates; (**D)** flow cytometric analysis of phenotypic differentiation during maturation of the thymic CD10^+^ PD-1^+^ and CD10^−^ PD-1^–^ population. Data are representative of at least two biological replicates.

TCRαβ^+^ CD4^–^ CD8α^+^ CD10^+^ PD-1^+^ T cells were sorted from human postnatal thymus to assess their proliferative capacity in the presence of interleukin-7 (IL-7) or interleukin-15 (IL-15), 2 cytokines reported to be present in peripheral tissues [14,15]. As controls, conventional TCRαβ^+^ CD4^–^ CD8α^+^ CD10^–^ PD-1^–^ T cells and unconventional TCRγδ^+^ T cells were included. During the first six days of culture, cell death was prominent in all conditions. Within ten days, the number of viable conventional CD10^−^ PD-1^–^ T cells had decreased even further. However, the TCRγδ^+^ and the CD10^+^ PD-1^+^ population started to expand markedly with IL-15 (Figure S1). This suggested that the CD10^+^ PD-1^+^ population was heterogeneous and possibly contained a significant fraction of IL-15-unresponsive immature cells. As CD1a is previously identified as an immaturity marker highly expressed on immature conventional T cells, while the CD1a^–^ phenotype is associated with maturity [16], CD1a expression by the CD10^+^ PD-1^+^ T cells was analyzed (Figure 1A). It was shown that the CD10^+^ PD-1^+^ population mainly consisted of CD1a^+^ cells and that this CD1a^+^ fraction was significantly larger than in the conventional T cell population (Figure 1B). Subsequently, CD10^+^ PD-1^+^ CD1a^+^ and CD10^+^ PD-1^+^ CD1a^–^ cells were sorted and cultured separately in the presence of IL-15. It was clear that the CD1a^+^ cells rapidly died in these cultures, while the CD1a^−^ fraction survived and showed homogeneous proliferation, as illustrated by CellTrace Violet dye dilution (Figure 1C). This indicated that solely the CD10^+^ PD-1^+^ CD1a^–^ cells are functionally mature thymocytes and are capable of surviving and expanding after emigration from the human thymus. The maturation marker CD1a could therefore be used in multicolor flow cytometry to evaluate the phenotypic changes of the CD10^+^ PD-1^+^ population during thymic maturation (Figure 1D). Expression of the early maturation markers CD27 and CD69 in relation to CD1a by the CD10^+^ PD-1^+^ population was different compared to the conventional expression pattern of the CD10^-^ PD-1^-^ thymocytes. Immature CD10^+^ PD-1^+^ cells showed a markedly higher CD28 expression than conventional thymocytes, possibly reflecting the earlier finding that the CD10^+^ PD-1^+^ population descends from a CD8αα^+^ PD-1^+^ CD28^+^ double positive blast progenitor early after β-selection [11]. During maturation, however, CD28 expression was rapidly downregulated in the CD10^+^ PD-1^+^ population, whereas the conventional population showed a stable CD28 expression. In addition, while conventional T cells obtained CCR7 and high levels of CD6 expression upon terminal maturation, the CD10^+^ PD-1^+^ cells remained CCR7 negative and dimly expressed CD6 upon maturation. Finally, the Fas receptor CD95, which is expressed by activated as well as by stem cell memory T cells [17], became expressed during maturation of CD10^+^ PD-1^+^ cells. The absence of CD6, CCR7, and CD28 combined with the presence of CD95 might be an important phenotypic characteristic defining this lineage after exiting the thymus.

### 2.2. IL-15 Induces Innate and Effector Characteristics in the CD10^+^ PD-1^+^ IELp Population.

**Figure 2:**
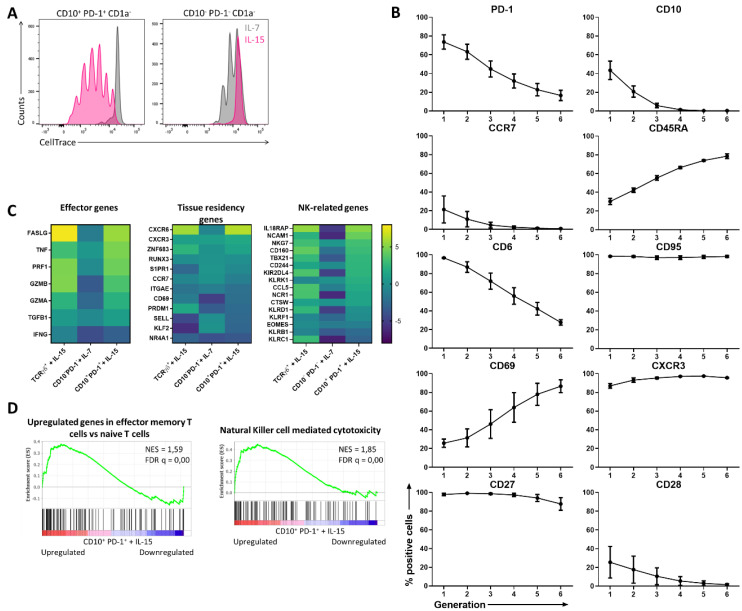
The proliferation of CD10^+^ PD-1^+^ IELps with IL-15 induces innate and effector characteristics. (**A**) Proliferation assessed by CellTrace Violet dye dilution in IELps and conventional T cells isolated from the human postnatal thymus, after 5 days of incubation in the presence of IL-7 (10 ng/mL) or IL-15 (10 ng/mL). Representatives of at least five experiments; (**B**) phenotypical changes of the IELps during proliferation with IL-15, measured using flow cytometry and plotted per generation (Figure S2) (mean ± SEM, n = at least 2); (**C**) heatmap for log2 fold change for selected genes, including effector genes, tissue residency genes, and NK-related genes, expressed in TCRγδ^+^ cells incubated with IL-15, conventional T cells with IL-7 and IELps with IL-15 for 11 days, compared to the same population freshly isolated from human postnatal thymus at day 0; (**D**) Gene Set Enrichment Analysis (GSEA) on IELps proliferated with IL-15 compared to freshly sorted IELps cells from human thymus. Left: genes upregulated in effector memory T cells compared to naive T cells as described by Gattinoni et al. [21]. Right: KEGG gene set of genes associated with NK-mediated cytotoxicity (hsa04650). Normalized enrichment score (NES) and false discovery rate q value (FDR q) are shown.

As it was shown that only the mature CD1a^–^ fraction of the investigated populations had proliferative capacity, the following experiments only include the mature CD1a^−^ fraction of the CD10^–^ PD-1^–^ (hereinafter referred to as conventional T cells) and the CD10^+^ PD-1^+^ IELp population (referred to as IELps), even if not specifically noted. When comparing the cytokine-driven proliferation of conventional T cells and the IELp population using CellTrace dye dilution assays, it was confirmed that IELps extensively proliferated only with IL-15, contrary to conventional T cells proliferating to a limited extent with IL-7 and incapable of proliferating with IL-15 (Figure 2A). It was evidenced in mice that IL-15 is not obligatory for thymic IELp development, but can promote IELp maturation and expansion and regulated homeostasis of CD8αα^+^ IELs in the murine intestine [18,19]. As it was hypothesized that proliferation in the presence of IL-15 also reflected the human extrathymic situation, the phenotypical changes occurring in the progeny of IELps were analyzed. CellTrace dye dilution was used to evaluate phenotypical changes of IELps during proliferation with IL-15 (Figure S2). Expression of CD10 rapidly declined during proliferation and PD-1 expression simultaneously became weaker. This validated our approach, as the corresponding population in human cord blood does not express CD10 (even though membrane metalloendopeptidase (*MME*) mRNA encoding CD10 is still upregulated) and only dimly expresses PD-1 compared to the thymic population [11]. The ability to downregulate PD-1 expression was also observed for the murine type A IELps [8]. CCR7, CD28, and CD6 expression further declined, while the surface expression of CD45RA and CD69 was sharply upregulated. The IELps maintained a stable, high expression of CD95, CD27, and CXCR3 (Figure 2B). The obtained CCR7^−^ CD45RA^+^ phenotype resembled the terminally differentiated effector memory T cells re-expressing CD45RA (TEMRA) already described in adult human peripheral blood [20]. Similar to the IELp population, the peripheral blood TEMRA population found in adults expressed CD95. However, TEMRAs can be distinguished from IELps as the TEMRA population is homogeneously CD69^−^ CD6^+^ and only partially CD28^−^ and CD27^+^ (Figure S3).

To further evaluate changes induced by cytokine-driven proliferation, RNA-sequencing was performed on the TCRγδ^+^ population cultured with IL-15, the conventional T cell population cultured with IL-7, and the IELp population cultured with IL-15 and each compared to the same population freshly isolated from human postnatal thymus. Overall, the IELp population revealed similar characteristics as the innate TCRγδ^+^ population, while the conventional population preserved its resting phenotype with IL-7 (Figure 2C). Effector genes such as *FASLG*, *TNF*, *PRF1*, and *GZMB* were markedly upregulated in the IELp population under influence of IL-15, while interferon gamma (*IFNG*) showed no clear expression in neither of the three populations (Figure 2C). Gene Set Enrichment Analysis (GSEA) revealed that genes upregulated in human effector memory T cells, as described by Gattinoni et al. [21], were significantly enriched in IELps after culture with IL-15 (Figure 2D). Natural killer (NK)-related genes such as IL-18 receptor accessory protein (*IL18RAP*), *NCAM1*, *NKG7*, and several NK receptors were also notably upregulated after culture with IL-15 (Figure 2C), along with a significantly enriched KEGG gene set on NK cell-mediated cytotoxicity (Figure 2D). This suggests that IELps in the tissues are likely more activated and exhibit NK-like functionality. Contrary to conventional T cells, IELps upregulated the chemokine receptors *CXCR6* and *CXCR3*, while the lymph node-homing markers *CCR7* and *SELL* were downregulated (Figure 2C). This might indicate a preference of IELps for homing to the peripheral tissues instead of the lymphoid organs. In conclusion, these data indicate that IL-15 plays an important role in maintaining and activating IELps, resulting in proliferation and upregulation of innate-like and cytotoxic genes.

The recently published cell atlas for the human thymus mainly subdivides the unconventional CD8αα^+^ TCRαβ^+^ T cells into *GNG4*^+^ CD8αα^+^ T(I) and *ZNF683*^+^ *MME*^+^ CD8αα^+^ T(II) cells sharing *PDCD1* expression, the gene encoding for PD-1 [22]. While our paper focuses on the CD8αα^+^ T(II) cells expressing *MME*, it was striking that the most downregulated gene of the IELp population following proliferation with IL-15 was *GNG4*, suggesting that this subpopulation does not proliferate in the presence of IL-15 (Figure S4).

### 2.3. The CD10^+^ PD-1^+^ IELp Population Gains a TCR-Independent Innate-Like Cytotoxic Functionality in Response to IL-15, Further Augmented by Inflammatory Cytokines

**Figure 3:**
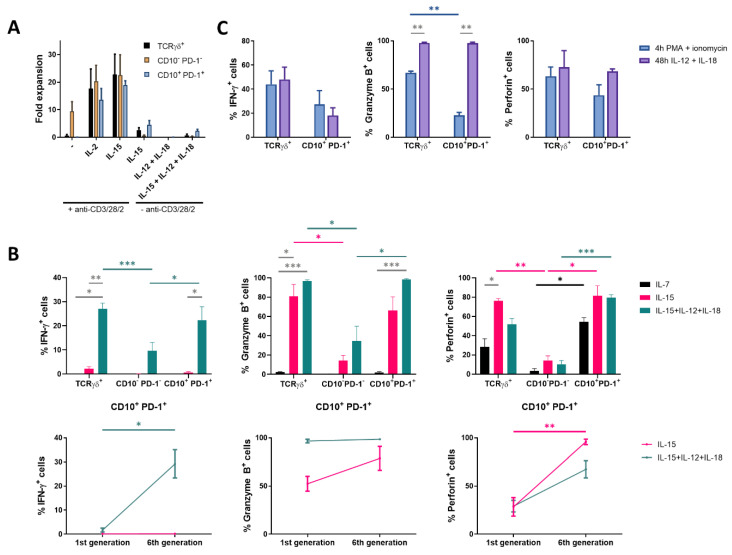
Proliferation with IL-15 induces cytotoxic mediators in CD10^+^ PD-1^+^ IELps. (**A**) fold expansion of TCRγδ^+^, conventional and IELp cells cultured for 8 days under various conditions (n = 2); (**B**) IFN-γ, granzyme B and perforin production by unstimulated TCRγδ^+^, conventional and IELp cells incubated for 5 days in the presence of IL-7, IL-15 or IL-15 + IL-12 + IL-18. IFN-γ, granzyme B, and perforin production measured via intracellular flow cytometry. Bottom: Cytokine production in IELps, plotted for the first and sixth/last generation (Figure S5; mean ± SEM, n = 3); (**C**) flow cytometric analysis of IFN-γ, granzyme B and perforin production by TCRγδ^+^ cells and IELps proliferated for 6 days with IL-15, followed by stimulation with IL-12 + IL-18 for 48 h or with PMA + ionomycin for 4 h (mean ± SEM, n = 3). Dunnett’s multiple comparisons test was used to assess statistically significant differences in cytokine production between the different populations and different interleukins. *p*-value < 0.05 (*), *p* < 0.01 (**), and *p* < 0.001 (***).

Above, it was shown that IL-15 induced proliferation as well as expression of genes involved in cytotoxicity in CD10^+^ PD-1^+^ IELp cells. To further assess its functionality, the IELp population was stimulated through the TCR. Unlike conventional T cells, the IELp population was incapable of proliferation following TCR stimulation and co-stimulatory signals (anti-CD3/CD28/CD2) (Figure 3A). This could be explained by the absence of CD28 expression on mature IELps (Figure 1D). TCR stimulation in the presence of cytokines induced expansion in the IELp population. In contrast, IELps expanded with IL-15 alone or combined with the inflammatory cytokines interleukin-12 (IL-12) and interleukin-18 (IL-18), a characteristic not seen for conventional T cells (Figure 3A).

To confirm the RNA-sequencing data (Figure 2C) at a protein level, production of the effector cytokine interferon-γ (IFN-γ) associated with antiproliferative, pro-apoptotic, and antitumor mechanisms, the serine protease granzyme B and the pore-forming protein perforin following culture with IL-7 or IL-15 was measured by intracellular flow cytometric analysis. In the presence of IL-15, IELps showed a high granzyme B and perforin production, two key granule proteins required for efficient cytotoxic activity, while almost no IFN-γ was produced (Figure 3B). As the IELp population showed NK-related characteristics after culture with IL-15 (Figure 2C–D), the inflammatory cytokines IL-12 and IL-18, which induce production of IFN-γ by NK cells [23], were added to the IELp cultures. Indeed, IL-15 combined with IL-12 and IL-18 significantly increased IFN-γ production in the IELp population. Furthermore, IFN-γ production significantly increased with each cell division, visualized by CellTrace dye dilution (Figure 3B and Figure S5). The addition of IL-12 and IL-18 had no significant influence on both granzyme B and perforin production, which were already highly expressed with solely IL-15 (Figure 3B). Although IFN-γ and perforin production by the IELp population increased as the cells proliferated in the presence of IL-15 + IL-12 + IL-18, granzyme B production was already maximal before cell division started (Figure 3B). Although the few viable conventional cells remaining after culture with IL-15 + IL-12 + IL-18 produced some IFN-γ, granzyme B, and perforin, the production was similar as seen after culture in the presence of IL-7 or IL-15 and significantly lower when compared to the IELp population. In general, cytokine production by the IELp population under the influence of different cytokines was comparable to the innate TCRγδ^+^ population (Figure 3B).

Finally, cytokine production after a short period of stimulation was assessed. Therefore, IELps were first incubated with only IL-15, followed by stimulation with IL-12 + IL-18 for 48 h or with phorbol myristate acetate (PMA) + ionomycin for 4 h, to circumvent the T cell membrane receptor complex, as no expansion was observed upon anti-CD3/CD28/CD2 stimulation alone (Figure 3A). Proliferation with IL-15 followed by IL-12 + IL-18 stimulation again resulted in similar intracellular cytokine production by the IELp and the innate TCRγδ^+^ population. No significant differences in IFN-γ or perforin production could be demonstrated for either population after IL-12 + IL-18 or after PMA + ionomycin stimulation (Figure 3C). However, stimulation with IL-12 + IL-18 resulted in a significantly higher granzyme B production as compared to PMA + ionomycin stimulation for both populations (Figure 3C). No major differences in cytokine production were observed between the cultures first proliferated with IL-15 and consecutively shortly stimulated with IL-12 + IL-18 or the cultures simultaneously combining IL-15 + IL-12 + IL-18 stimulation (Figure 3B,C). 

Taken together, the IELp population seemed to be less dependent on TCR-mediated activation but rather became functionally active in the presence of IL-15, further augmented by the inflammatory cytokines IL-12 and IL-18. Under these conditions, IELps acquired cytotoxic potential and cytokine production. These characteristics were also described for human unconventional MAIT cells, in addition to their TCR-dependent mechanisms [24,25]. 

Further exploration of the specificity of the human IELps is required. Although it is suggested that autoantigens are the ligands of their TCRs [6], the nature of these autoantigens and their condition of expression remains unknown. These autoantigens may be only expressed under particular conditions, such as cellular stress. The importance of TCR stimulation in thymic IELp development is evidenced in mice [5,6,19] as well as in humans [11], but the role of the TCR in the maintenance or activation of IELps remains ambiguous. The IELp population failed to expand upon TCR stimulation alone, while the combination with interleukins resulted in more pronounced expansion, even compared to culture with solely interleukins. In addition, stimulation with PMA + ionomycin, activating several intracellular signaling pathways while bypassing TCR and CD28 costimulatory signaling, resulted in T cell activation and production of cytokines. Generally, the contribution of this innate-like cytotoxicity by IELps in the in vivo inflammatory immune response currently remains elusive. Based on these in vitro experiments, it appears as if IELps might augment the inflammatory response. As human IELps express PD-1 in the thymus, immune checkpoint inhibitors may likely interfere with their generation and subsequent migration to the gut. This may cause increased susceptibility to the endogenous gut flora with unwanted gut inflammation resulting in severe enteritis. This should be taken into account when PD-1 inhibitors are administered to children and young adults.

Our data provide clues regarding the phenotype of the progeny of human thymic IELps. As PD-1 and especially CD10 seem rather transient markers during IELp development, the CD95^+^ CXCR3^+^ CD6^−^ CCR7^−^ CD28^−^ phenotype defined in these data might advance future investigation of this lineage in human cord blood and tissues. As discussed above, it might be difficult to distinguish the TEMRA and the IELp lineage cells based on flow cytometry alone. Besides phenotypic markers, transcription factors may also be of added value in the identification of their progeny, as well as to investigate the mechanisms regulating human CD8αα T cell development. Specifically, Helios encoded by the *IKZF2* gene has been reported to be upregulated in human IELps [11,26].

## 3. Materials and Methods

### 3.1. Sample Processing

Human postnatal thymus was obtained from children who underwent cardiac surgery, peripheral blood was obtained from healthy adult blood donors. Samples were used following the guidelines of the Medical Ethical Committee of Ghent University Hospital (CG20171208A, 8 December 2017) after informed consent had been obtained in accordance with the Declaration of Helsinki. Mononuclear cells were isolated from thymus single-cell suspensions and peripheral blood buffy coats using density gradient centrifugation (LymphoPrep; Axis-Shield, Dundee, UK; 1114547). Thymus suspension was enriched for CD8^+^ cells by magnetically activated cell sorting (MACS) through negative selection using anti-CD4-biotin (homemade) alone (Figure S1) or in combination with anti-CD1-biotin (homemade) and anti-biotin Microbeads (Miltenyi Biotec, Leiden, The Netherlands; 130-090-485).

### 3.2. Flow Cytometry and Antibodies

Staining of surface markers was performed in DPBS (Lonza, Basel, Switzerland; 17-512F) with 1% fetal calf serum (FCS; Biowest, Nuaillé, France; S1810) using the antibody to cell ratio recommended by the supplier. Intracellular staining was performed following the supplier’s protocol using BD Cytofix&Cytoperm (BD Biosciences, Erebodegem, Belgium; 554714). Flow cytometric analysis was performed on the LSR II and cell sorting on the ARIA II (both BD Biosciences). Viable cells were gated based on propidium iodide (PI) negativity or Fixable Viability Dye (eFluor 506; Thermo Fisher Scientific, Merelbeke, Belgium; 65-0866-18) negativity for surface and intracellular stainings respectively. The following list of anti-human monoclonal antibodies was used. Fluorescein isothiocyanate (FITC)-conjugated: CD45RA (homemade), CD69 (Biolegend, London, UK; 310904), IFN-γ (BD Biosciences, 554551); phycoerythrin (PE)-conjugated: PD-1 (CD279, Biolegend, 367404), CD95 (Biolegend, 305608), Granzyme B (eBioscience, Vienna, Austria; 12-8899-42), Perforin (eBioscience, 12-9994-42), Streptavidin (BD Biosciences, 349023); allophycocyanin (APC)-conjugated: CD28 (Miltenyi, 130-092-923), CXCR3 (Biolegend, 353707), TCRγδ (Miltenyi, 130-113-500); Brilliant Violet 421-conjugated: CD1a (Biolegend, 300128); PE Cy7-conjugated: CD10 (Biolegend, 312214); APC Cy7/APC Fire750-conjugated: CD8α (Biolegend, 344746), CD27 (Biolegend, 302816), CCR7 (CD197, Biolegend, 353246); peridinin chlorophyll protein complex (PerCP) Cy5.5-conjugated: CD4 (Biolegend, 344608); biotin-conjugated: CD6 (Miltenyi, 130-098-766). CD4/CD1-depleted thymus suspension was sorted into TCRγδ^+^ (TCRγδ^+^ population), TCRγδ^-^ CD8α^+^ CD10^-^ PD-1^-^ (CD10^-^ PD-1^-^ population) and TCRγδ^-^ CD8α^+^ CD10^+^ PD-1^+^ (CD10^+^ PD-1^+^ population) and further cultured in IMDM (Thermo Fisher Scientific, 12440053) supplemented with 10% FCS, 2 mM L-glutamine (Thermo Fisher Scientific, 25030-081), 100 IU/mL penicillin and 100 IU/mL streptomycin (Thermo Fisher Scientific, 15140-122) (complete IMDM, cIMDM).

### 3.3. RNA-Sequencing

The TCRγδ^+^ population and the CD10^+^ PD-1^+^ population were cultured in cIMDM supplemented with IL-15 (10 ng/mL; Miltenyi, 130-095-765) and the CD10^−^ PD-1^−^ population in cIMDM supplemented with IL-7 (10 ng/mL; Miltenyi, 130-095-362) for 11 days before the cells were harvested for transcriptomics analysis. RNA extraction was performed using the miRNeasy Mini Kit (Qiagen, Venlo, The Netherlands; 217004). For poly(A) RNA-seq, the QuantSeq 3′ mRNA FWD kit (Lexogen, Vienna, Austria) was used, followed by single-ended sequencing on the NextSeq500 Sequencing System (Illumina, San Diego, CA, USA) with a read length of 75bp. RNA-seq reads were aligned to hg38-noalt using STAR v2.6.0c and quantified on Ensembl v93.

### 3.4. Expansion Assay

For each sorted population, 1 × 10^5^ cells were seeded per well in a 96-well culture plate (BD Biosciences) in 100 µL cIMDM supplemented with ImmunoCult Human CD3/CD28/CD2 T Cell Activator (StemCell Technologies, Vancouver, Canada; 10970) alone, ImmunoCult + IL-2 (10 ng/mL; Miltenyi, 130-097-748), Immunocult + IL-15 (10 ng/mL); or cytokines alone, without Immunocult: IL-15 (10 ng/mL), IL-12 (10 ng/mL; PeproTech, Hamburg, Germany; 200-12) + IL-18 (10 ng/mL; R&D Systems, Minneapolis, MN, USA) or IL-15 + IL-12 + IL-18 (all 10 ng/mL). ImmunoCult was added as described by the manufacturer. After 8 days, viable cell count was determined using flow cytometry.

### 3.5. CellTrace Proliferation Assays

For each sorted population, the cells were labeled with the CellTrace Violet Cell Proliferation Kit (Invitrogen, Merelbeke, Belgium; C34557) following the standard protocol for labeling cells in suspension as described by the manufacturer. Afterwards, 1 *×* 10^5^ cells were seeded per well in a 96-well culture plate in 100 µL cIMDM supplemented with IL-7 (10 ng/mL), IL-15 (10 ng/mL) or IL-15 + IL-12 + IL-18 (each 10 ng/mL). For intracellular cytokine production induced by proliferation, brefeldin A (Golgiplug; BD Biosciences, 555029) was added during the last 4 h of culture. Hereafter the cells were fixed, permeabilized, and stained for IFN-γ, granzyme B, and perforin as described above.

### 3.6. Stimulation Assay

The TCRγδ^+^ population and CD10^+^ PD-1^+^ population were cultured with IL-15 for 6 days after which they were stimulated by the addition of IL-12 + IL-18 (both 10 ng/mL) for 48 h. Brefeldin A was added during the last 4 h. On the other hand, the TCRγδ^+^ population and CD10^+^ PD-1^+^ population were cultured with IL-15 for 7 days and 20 h before they were stimulated by the addition of PMA (1 ng/mL; Sigma-Aldrich, Diegem, Belgium; 16561-29-8) and ionomycin (0.5 µg/mL; Sigma-Aldrich, 56092-82-1) for 4 h, during which also brefeldin A was added. After stimulation, the cells were harvested, fixed, permeabilized, and stained for IFN-γ, granzyme B, and perforin as described above.

### 3.7. Statistical Analysis

Statistical analyses were performed in Prism version 8.4.3. (GraphPad Software, San Diego, CA, USA), using statistical tests as indicated in figure legends. Results were considered statistically significant when the *p*-value was less than 0.05. GSEA was performed using the GSEA software version 4.0.3, a joint project of UC San Diego (San Diego, CA, USA) and Broad Institute (Cambridge, MA, USA) [27,28]. The GSEAPreranked tool was run using standard parameters and 1000 permutations. The gene list was ranked by comparing the CD10^+^ PD-1^+^ IELp population cultured in the presence of IL-15 for 11 days to the freshly sorted CD10^+^ PD-1^+^ cells from human thymus, ranked from the upregulated genes (left) to the downregulated genes (right) due to IL-15. The normalized enrichment score (NES) reflects the degree to which the gene set is overrepresented in the upregulated genes (positive value) or downregulated genes (negative value). The false discovery rate q value (FDR q) is the estimated probability that a gene set with a given NES represents a false-positive finding.

## Supplementary Materials

Supplementary Materials can be found at https://www.mdpi.com/1422-0067/21/22/8785/s1.

## Abbreviations

GSEA	Gene set enrichment analysis
IEL	Intraepithelial lymphocyte
IELp	Intraepithelial lymphocyte precursor
IFN-γ	Interferon-γ
IL-7	Interleukin-7
IL-12	Interleukin-12
IL-15	Interleukin-15
IL-18	Interleukin-18
MAIT	Mucosal-associated invariant T cell
MME	Membrane metalloendopeptidase
NK	Natural killer
PMA	Phorbol myristate acetate
TCR	T cell receptor
TEMRA	Effector memory T cells re-expressing CD45RA
