# Supplementary material for: Human Thymic CD10+ PD-1+ Intraepithelial Lymphocyte Precursors Acquire Interleukin-15 Responsiveness at the CD1a– CD95+ CD28– CCR7– Developmental Stage
Source: Int J Mol Sci. 2020 Nov 20;21(22):8785. doi: 10.3390/ijms21228785 (PMC7699974; doi:10.3390/ijms21228785)
Supplement: Supplementary file 1 [file ijms-21-08785-s001.pdf]

International Journal of
Molecular Sciences

MDPI

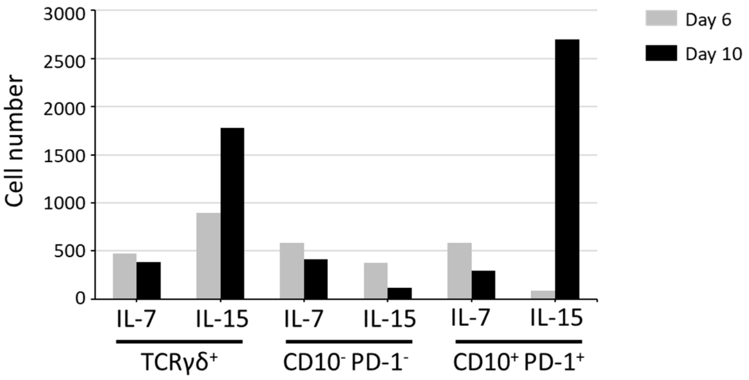

Supplementary Figure 1. A total of 25 x 10^3 cells TCRγδ$^+$ cells, CD10$^-$ PD-1$^-$ cells and CD10$^+$ PD-1$^+$ cells (without taking into account CD1a expression) were cultured in the presence of IL-7 (10 ng/mL) or IL-15 (10 ng/mL) and assayed for viable cell count at day 6 and day 10 (n = 1).

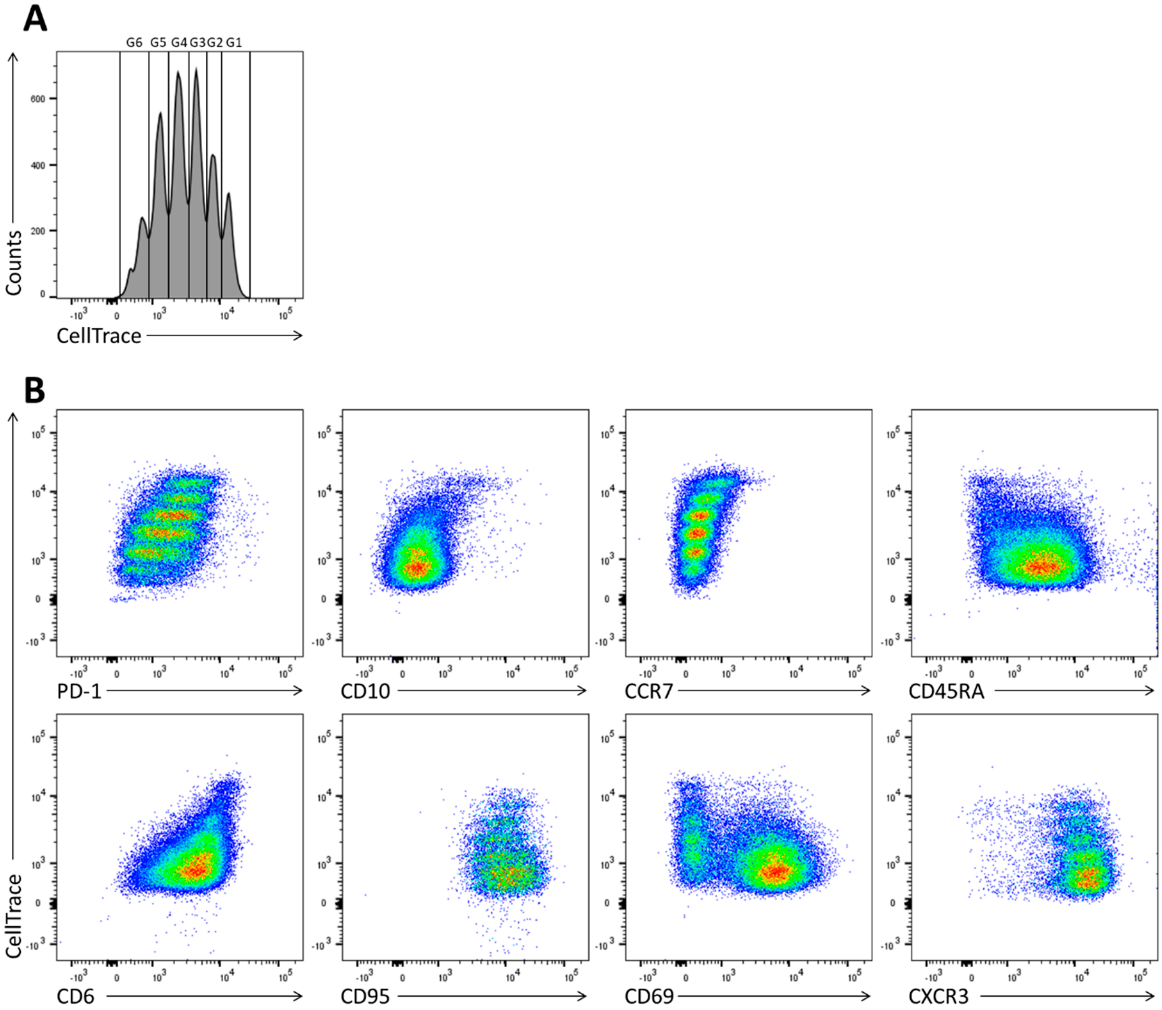

Supplementary Figure 2. Flow cytometric analysis of the CD10$^+$ PD-1$^+$ IELp cells during proliferation with IL-15. (**A**) Gating strategy for the six generations, indicating successive rounds of cell division. Proliferation visualized by CellTrace Violet dye dilution from right to left. (**B**) Dot-plots of the phenotypical markers presented in figure 2B.

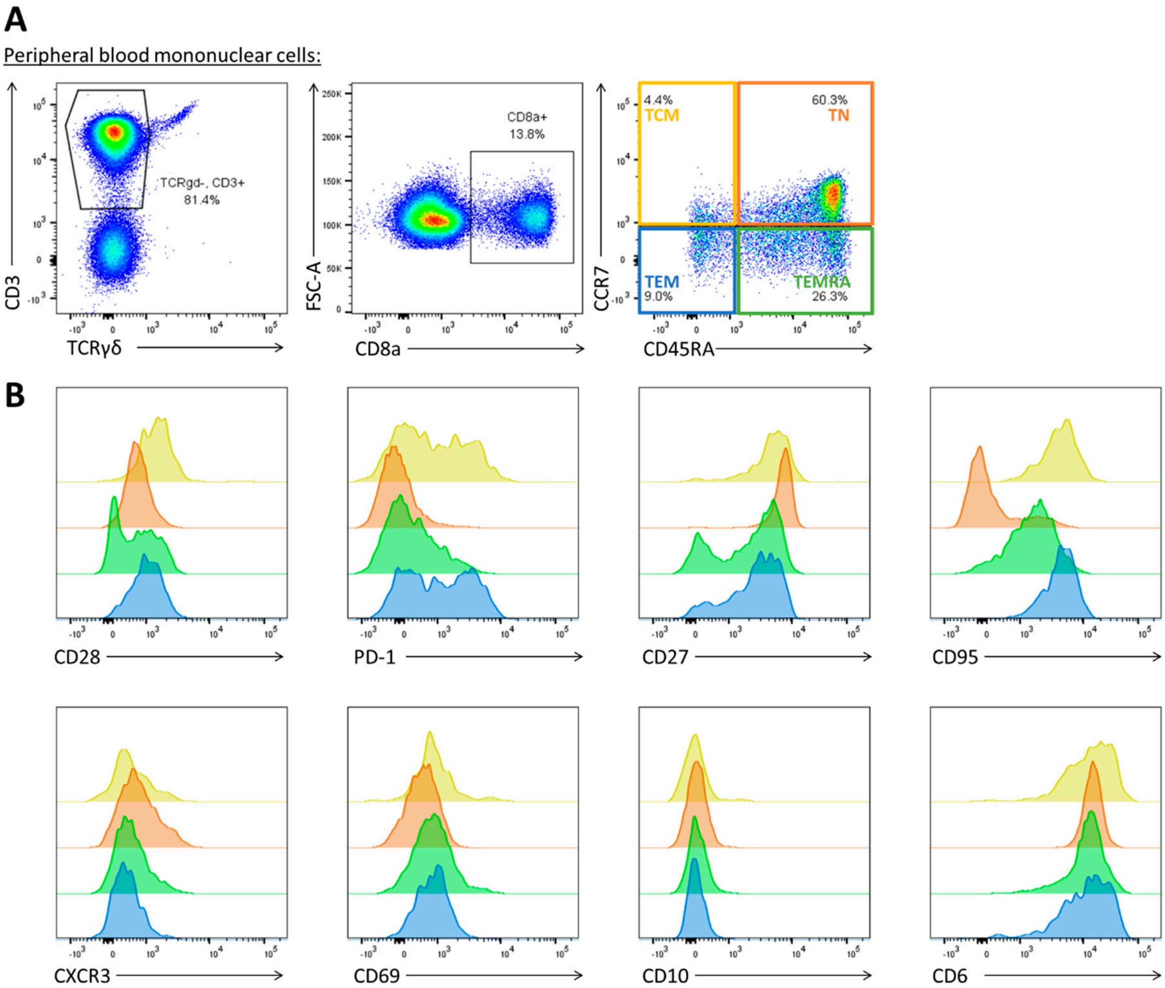

Supplementary Figure 3. Phenotypic comparison of central memory (TCM), naive (TN), effector memory re-expressing CD45RA (TEMRA) and effector memory (TEM) T cells. (**A**) Gating strategy for $CD45RA^-CCR7^+$ TCM (yellow), $CD45RA^+CCR7^+$ TN (orange), $CD45RA^+CCR7^-$ TEMRA (green) and $CD45RA^-CCR7^-$ TEM (blue) from peripheral blood from healthy adult donors. (**B**) Histograms showing the expression of phenotypical markers in TCM (yellow), TN (orange), TEMRA (green) and TEM (blue). Representative of three biological replicates.

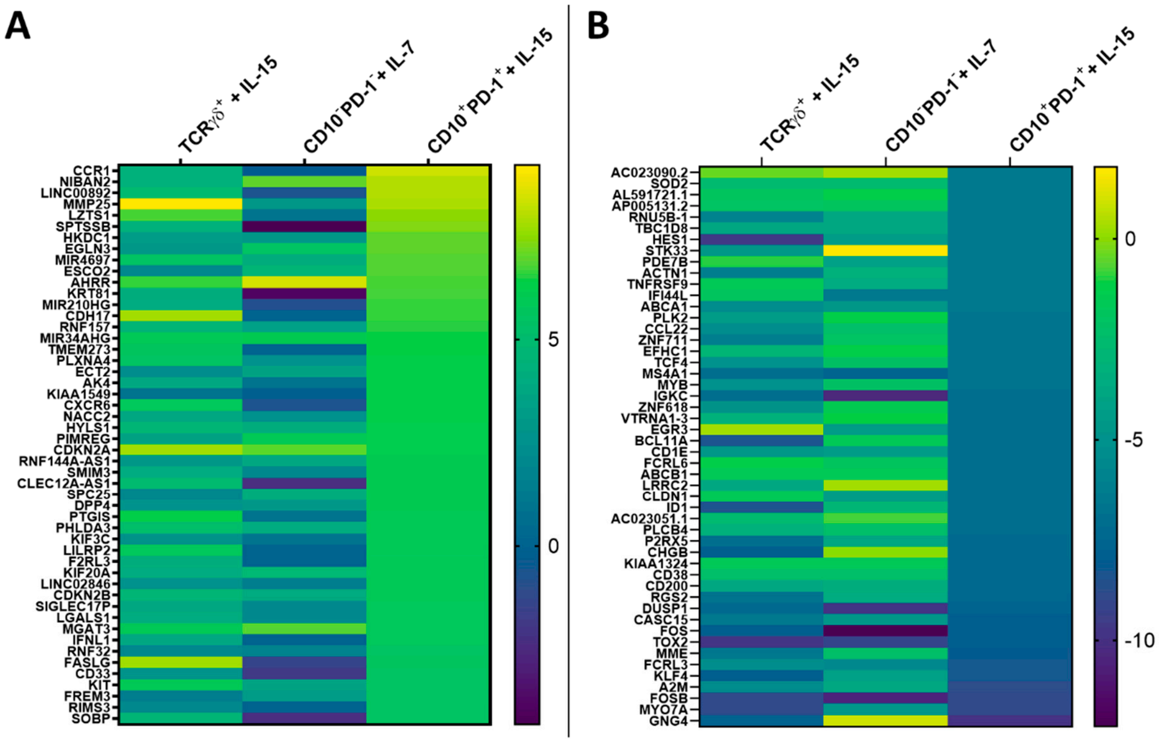

Supplementary Figure 4. Heatmaps for log2 fold change in expression in TCRγδ+ cells incubated with IL-15, CD10- PD-1- cells with IL-7 and CD10+ PD-1+ cells with IL-15 for 11 days, compared to the same populations freshly isolated from human postnatal thymus at day 0, showing (**A**) the top 50 most upregulated genes in CD10+ PD-1+ cells after culture with IL-15 and (**B**) the top 50 most downregulated genes in CD10+ PD-1+ cells after culture with IL-15.

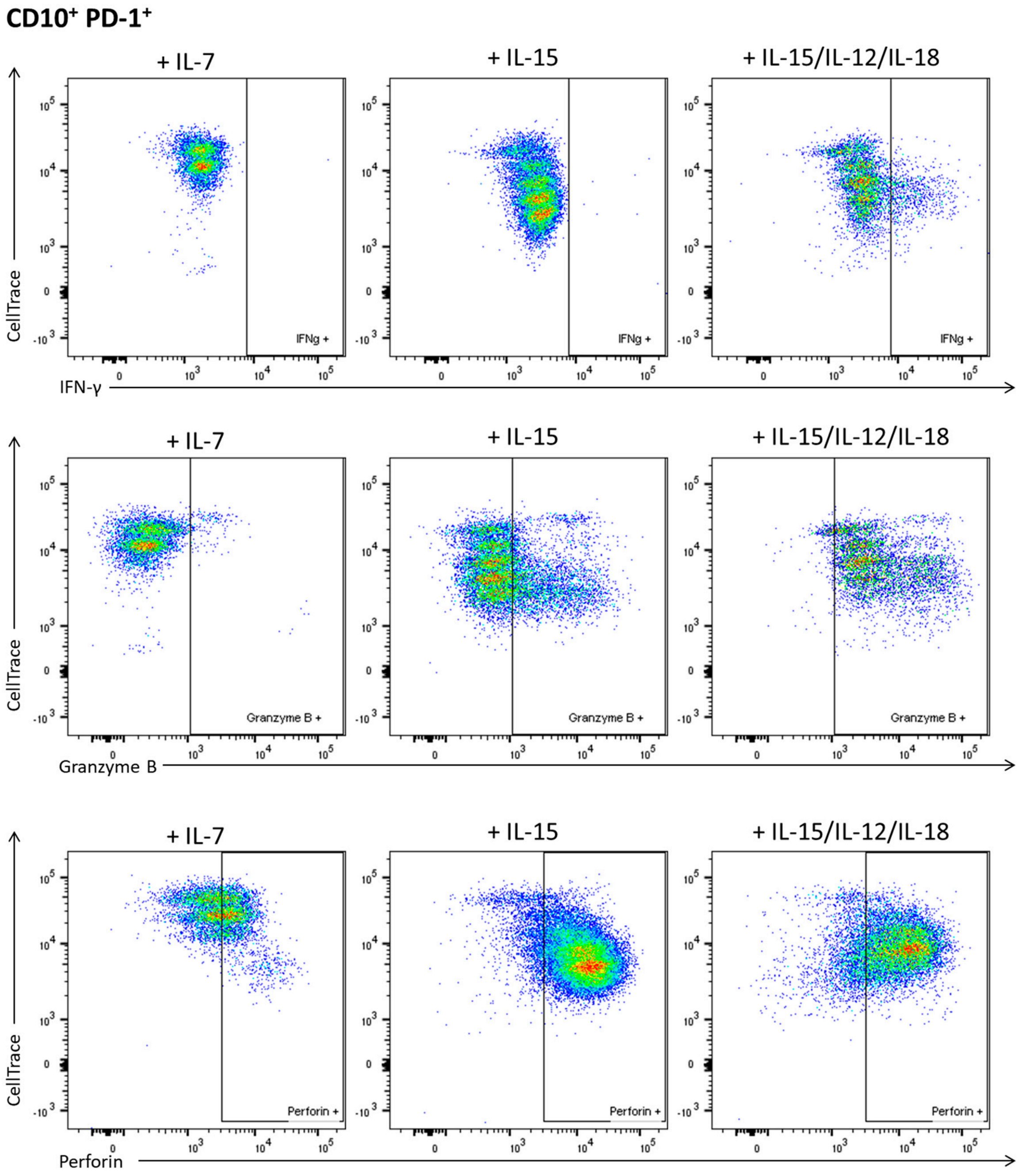

Supplementary Figure 5. Gating strategy used in figure 3B. Dot plots show the CD10$^+$ PD1$^+$ population proliferated in the presence of different interleukins for five days.
